# MALDI-TOF/MS Analysis of Non-Invasive Human Urine and Saliva Samples for the Identification of New Cancer Biomarkers

**DOI:** 10.3390/molecules27061925

**Published:** 2022-03-16

**Authors:** Carlo Zambonin, Antonella Aresta

**Affiliations:** Dipartimento di Chimica, Università degli Studi di Bari “Aldo Moro”, Via E. Orabona 4, 70124 Bari, Italy; antonellamaria.aresta@uniba.it

**Keywords:** MALDI-TOF/MS, cancer, biomarkers, early stage diagnosis, urine, saliva

## Abstract

Cancer represents a group of heterogeneous diseases that are a leading global cause of death. Even though mortality has decreased in the past thirty years for different reasons, most patients are still diagnosed at the advanced stage, with limited therapeutic choices and poor outcomes. Moreover, the majority of cancers are detected using invasive painful methods, such as endoscopic biopsy, making the development of non-invasive or minimally invasive methods for the discovery and fast detection of specific biomarkers a crucial need. Among body fluids, a valuable non-invasive alternative to tissue biopsy, the most accessible and least invasive are undoubtedly urine and saliva. They are easily retrievable complex fluids containing a large variety of endogenous compounds that may provide information on the physiological condition of the body. The combined analysis of these fluids with matrix-assisted laser desorption ionization–time-of-flight mass spectrometry (MALDI-TOF/MS), a reliable and easy-to-use instrumentation that provides information with relatively simple sample pretreatments, could represent the ideal option to rapidly achieve fast early stage diagnosis of tumors and their real-time monitoring. On this basis, the present review summarizes the recently reported applications relevant to the MALDI analysis of human urine and saliva samples.

## 1. Introduction

Cancer represents a large group of heterogeneous diseases that can affect any part of the body. It is a leading global cause of death, responsible for millions of deaths every year. Generally, the major cancer sites in terms of new cases are breast, lung, colon and rectum, prostate, skin, and stomach, while the sites responsible for the higher amounts of deaths are lung, colon and rectum, liver, stomach, and breast. The most recent estimates of the American Cancer Society [1] have predicted approximately 1,918,030 new cases and 609,360 deaths in the United States in 2022. The genesis of cancer depends on multiple mechanisms [2,3,4], a combined accumulation and functional cooperation between genetic and epigenetic changes that alter the control systems of the homeostatic balance between cell proliferation and death, leading to the transformation of healthy cells into malignant ones. These events are involved at every step, from responses to carcinogens exposure to the development of cancer. Since 1991, cancer mortality in the United States has dropped by 32% for different reasons, including reductions in smoking, treatment breakthroughs and, when possible, early detection through screening [1]. However, the fight against cancer remains a major challenge in public health that requires a multidisciplinary approach in different areas, such as understanding the mechanisms, incidence of risk factors, innovative treatment strategies, study of health disparities, new screening markers, and many others.

For nearly all tumors, management and survival are related to the stage, which is assigned according to location, cell type, size and spreading, even if this traditional classification does not entirely reflect the heterogeneous nature of cancer. Thus, tumors have been recently classified, by molecular subtyping studies, into more uniform subgroups characterized by common molecular features and clinical outcomes, in the attempt to obtain new and more effective diagnostic, prognostic, and therapeutic options [2]. However, many issues must be faced to successfully apply clinical applications of molecular subtyping. Advanced-stage tumors have poor survival rates, while better outcomes could be obtained if the disease is identified early, when it can be surgically removed or treated with milder therapies [5]. Unfortunately, cancer onset is generally associated with few to no symptoms and has many anatomic and molecular subtypes that require specific diagnostic approaches, meaning that most patients are diagnosed at the advanced stage. Furthermore, many individuals are reluctant to follow screening guidelines [6] and non-invasive early screening options are limited to the detection of few cancer types, in particular colon [7], prostate [8], breast [9], and cervical [10]. Most cancers are instead diagnosed using invasive painful methods, such as endoscopic biopsy, which consists of the microscope analysis of tissue surgically taken from the suspicious mass, that may cause further complications such as bleeding, inflammations, or infections [11].

It has become clear that non-invasive and reliable approaches for early-stage diagnosis and real-time monitoring of tumors based on known and/or newly discovered biomarkers are extremely advisable. Thus, an increasing interest has been observed towards body fluids such as blood, saliva, pleural fluid, peritoneal fluid, and brain spinal fluid, which constitute a valuable non-invasive alternative to tissue biopsy [12]. Among them, the most accessible and least invasive human specimens that can be considered are undoubtedly urine and saliva. Urine contains metabolites, proteins, peptides, and inorganic elements and could contain cancer-cell-derived molecules [13], thus having great potential as a source of biomarkers. It is inexpensive, easy to collect and handle, and available in large amounts. Even though urine is particularly suitable in the case of urological tumors, it may also provide information from other organs via plasma obtained through glomerular filtration [14]. Saliva is a complex fluid produced by the salivary glands and composed of large amounts of secretory proteins, electrolytes, and other age/sex-related substances, which has proved to be a suitable substrate for the detection different types of cancer [15,16]. It can also be easily retrieved and is suitable for repeated testing.

In addition to having samples obtained with non-invasive techniques, the relevant analyses should be performed using reliable instrumentation that allows information to be obtained quickly and with relatively simple sample pretreatment. For instance, several diagnostic applications in urine [17] and saliva [18] have been developed using mass spectrometry (MS) techniques. Among these, matrix-assisted laser desorption ionization–time-of-flight mass spectrometry (MALDI-TOF) represent a suitable option for obtaining valid and accurate data from complex samples [19], possessing many intrinsic advantages compared to other MS techniques. For instance, electrospray ionization (ESI) MS, which indeed provides more complete structural information than MALDI, must be directly coupled with liquid chromatography, making the separation process a major contributor to the analysis time. On the contrary, MALDI is a standalone instrument that permits to obtain mass spectra a few seconds after the introduction of the target plate, on which hundreds of separate samples can be applied simultaneously, after being prepared in parallel “offline”. Furthermore, MALDI analysis of complex samples can be performed directly or after clean-up/enrichment steps, permitting the determination of almost all molecular classes, including intact proteins, peptides, lipids, nucleotides and nucleosides, carbohydrates, and small molecular weight compounds. Furthermore, the instrumentation is usually easy to use even by unskilled personnel. All these feature makes MALDI the ideal technique for routine clinical analysis, as demonstrated by the huge number of existing applications for diagnostic purposes [19,20,21]. MALDI has indeed demonstrated a great potential when applied to the fight against cancer [22], and part of the huge amount of work performed on tumor-related topics, including the analysis of drugs [23], MALDI-imaging developments [24,25], diagnosis of ovarian [26], non-small cell lung [27], and prostate [28] cancer, has very recently been reviewed.

Since non-invasive or minimally invasive early diagnosis of cancer is becoming increasingly important, the present work aims to review the MALDI applications dedicated to the detection and identification of novel potential cancer biomarkers reported in the recent scientific literature (from 2010 to date) and performed by analyzing non-invasive specimens, namely urine and saliva samples.

## 2. Urine Samples Analysis

### 2.1. Prostate Cancer

Prostate cancer (PrC) is among the most frequent cancers worldwide [29] and the most diagnosed male malignancy in developed countries. The Serum Prostate-Specific Antigen (PSA) measurement is the current screening procedure for its detection and surveillance. Since its introduction, a significant reduction in prostate cancer mortality has been observed, but the test is characterized by high false positive rate [30] due to a poor diagnostic accuracy, leading to overdiagnosis and overtreatment [31]. Therefore, patients with increased PSA values still need to confirm the presence of cancer undergoing prostate biopsy, and the search for new biomarkers that are sufficiently reliable to be used in clinical practice is always underway.

A MALDI-TOF proteomic study was performed on human urine to study the influence of different variables (sample collection, storage, preparation, and deposition on the target plate) on the proteins/peptides observed in the MS spectra [32]. The best spectra in terms of number and abundance of detected ions and reproducible profiles were obtained using a specific workflow, involving short handling times, low storage temperatures of acidified and centrifuged samples, home-made microcolumns packed with hydrophilic–lipophilic balance resin for sample extraction, and spin coating for sample deposition on the target plate. In these conditions, some signals potentially related to the disease were only observed in PrC patients. However, a complete characterization was not possible due to intrinsic limitation of the MALDI used and further investigation by MS/MS techniques would have been necessary to confirm their sequence and origin. By comparison with literature data, only the *m*/*z* ion 1432.91 was hypothesized to be a peptide probably originating from semenogelin I isoform b preprotein. The improvement of data reliability, together with the search for new biomarkers, was also the aim of a further MALDI based peptidomic study conducted on post-prostatic massage urine and serum [33] of healthy donors and patients with lower urinary tract symptoms. Different experimental strategies were evaluated, and the most feasible approaches eventually adopted. Even though several peptides were shared by urine and serum, only few serum features exhibited statistical significance, indicating that serum peptidome was more effective than urine peptidome in PrC discrimination.

**Figure 1:**
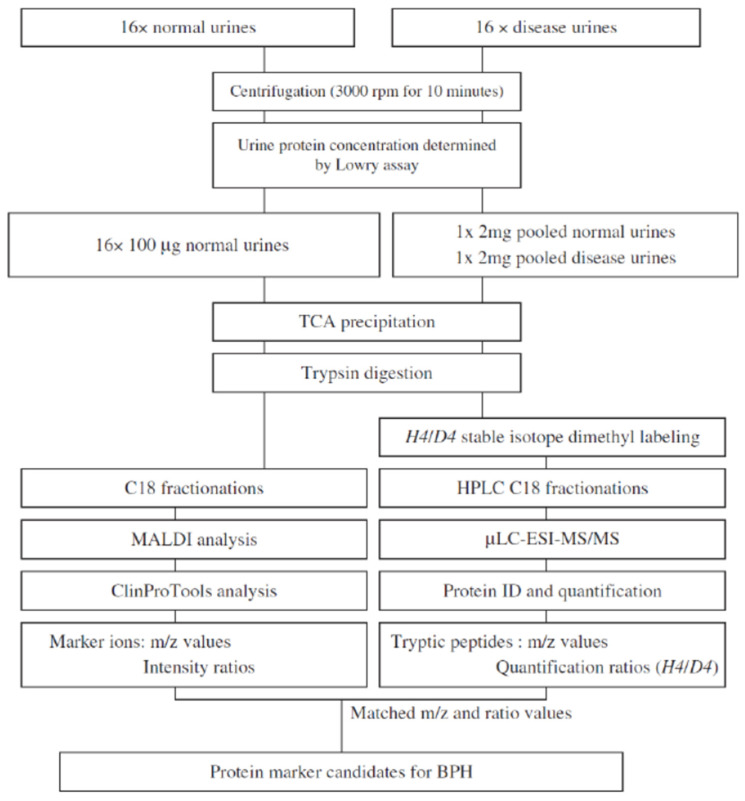
Schematics of the experimental workflow used in [34]. Reprinted with permission from Ref. [34]. Copyright 2011 John Wiley and Sons.

Another urinary proteomic study combined data obtained using MALDI-TOF, for the analysis of individual samples considering subject variations, and LC-ESI-MS/MS coupled with stable isotope labeling, for the analysis of normal and disease samples, which provides protein identity and quantification ratio, to search for specific biomarkers of benign prostatic hyperplasia that could differentiate patients affected by this pathology from both healthy and prostate cancer subjects [34]. Proteins in urine samples were digested into peptides by trypsin before analysis, following the workflow reported in Figure 1. Among nine potential markers identified, three underwent ELISA validation experiments and urinary CD14 was eventually identified as the most suitable for the purpose of the work.

**Figure 2:**
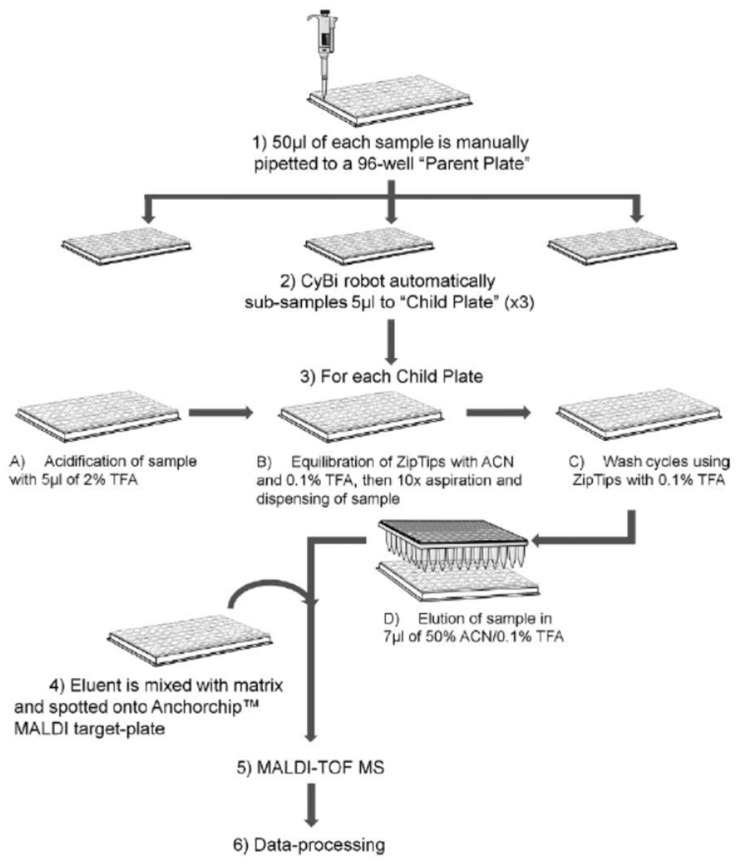
Schematic of the automated workflow adopted in [36]. Reprinted with permission from Ref. [36]. Copyright 2014 John Wiley and Sons.

A further application [35] carried out the MALDI-TOF/MS^n^ peptidomic analysis of urine samples of PrC patients and normal controls voided after digital rectal examination (DRE) with the specific aim of trying to overcome the diagnostic limits of PSA through the individuation of new markers for prostate cancer. Proteins/peptides in urine were captured, concentrated, and desalted by means of Sepharose ion-exchange resin, and subjected to MALDI analysis. The *m*/*z* ion 2331, attributed to a C-terminal PSA fragment composed of 19 amino acid residues, was indicated as a new potential pathognomonic biomarker for the detection of early signs of malignant transformation in the prostate. In another study, pre- and post-DRE urine samples, taken from prostate cancer patients and from individuals with benign prostatic conditions, were analyzed by MALDI following a simple automated workflow, reported in Figure 2, to determinate biomarkers able to differentiate between benign and malignant prostate diseases [36]. The *m*/*z* ions and their relative abundances were subjected to statistical analysis, and the *m*/*z* ion 10,760, attributed to β-microseminoprotein, was found to be significantly lower in the urine of cancer patients. Furthermore, the combination of β-microseminoprotein and PSA levels produced a high classification accuracy for PrC in the sample set under study. A proteomic study based on two-dimensional gel electrophoresis (2D-DIGE) followed by MALDI analysis identified aldehyde dehydrogenase 1A3 as a potential target of miR-187, a tumor suppressor microRNA downregulated in prostate cancer [37]. Then, urine samples taken post-DRE and prior to diagnostic needle biopsy from patients with suspicion of PrC were analyzed to evaluate aldehyde dehydrogenase 1A3 potential as a biomarker for prostate cancer, finding a positive association with tumor appearance.

A significant number of patients subjected to radical prostatectomy have positive surgical margins, an incomplete excision of malignant tissue that might imply additional treatments. Since it could be advisable to dispose of a fast a non-invasive mean to identify these risk patients, a pilot study evaluated the differences between the protein profiles of two experimental sets of urine samples, collected from cancer patients experiencing positive and negative surgical margins, respectively [38]. Differences in molecular weights of 80–99 and 150–235 kDa between the experimental groups were found by sodium dodecyl sulphate-polyacrylamide gel electrophoresis (SDS-PAGE). Moreover, peptide mass fingerprint of two-dimensional gel electrophoresis trypsin-digested spots analyzed by MALDI-TOF, highlighted the differences between expressed proteins. In total, three proteins known to have connections with PrC, cyclin-dependent kinase 6, galectin-3-binding protein and L-lactate dehydrogenase C chain, were found, only in the positive surgical margin set of samples, indicating their potential usefulness for a fast recognition of risk patients.

The established connection between Helicobacter pylori and stomach cancer has brought to the hypothesis that another microaerophylic organism, Propionibacterium acnes, could cause prostate cancer with a similar mechanism. Therefore, MALDI was applied to the analysis of post-prostate massage urine samples collected from active surveillance patients and controls, searching for aerobic and anaerobic bacteria [39]. Either microaerophilic and/or obligate anaerobic organisms were observed in a meaningful number of patients (41%), and the latter was never found in control samples. Interestingly, mean PSA was significantly higher in patients presenting microaerophylic bacteria.

Expressed prostatic secretions-urine samples, collected performing a gentle massage of the prostate gland during DRE prior to biopsy, prostatic fluids and urine were successfully analyzed by MALDI imaging to detect *N*-glycan profiles in view of new biomarkers discovery [40]. Glycoproteins were immobilized on an amine reactive slide and digested by *N*-glycosidase. The approach permitted to detect over 100 *N*-glycan compositions. The approach was potentially extendable to other liquid matrices, while analytes concentration could be increased by simply using more starting fluid.

Very recently, two different MALDI-TOF-based studies investigated the suitability of urinary lipids as potential biomarkers for prostate cancer. In the first work, samples from prostate cancer and benign prostatic hyperplasia were considered [41]. Urine samples collected after digital rectal examination were extracted using the acidified Bligh–Dyer method, and the levels of urinary lysophosphatidylcholine and phosphatidylcholines were compared, finding significant differences between PrC and benign prostatic hyperplasia patients. In a second study, the influence of experimental variables such as extraction procedure and target plate matrix were evaluated to optimize the MALDI-TOF analysis of lipids in urine samples obtained from PrC patients and healthy donors [42]. The best results were obtained using the Bligh–Dyer extraction and α-cyano-4-hydroxycinnamic acid matrix. Lysophosphatidylcholine, phosphatidylcholine, phosphatidylethanolamine, phosphatidylinositol, and triacylglycerols were found in urine samples, permitting the discrimination between the two groups with good accuracy.

### 2.2. Bladder Cancer

Bladder cancer (BC) is the tenth most common cancer worldwide. Most cases of BC are non-muscle invasive (NMIBC), while the remaining ones are muscle invasive (MIBC) [43]. The available diagnostic techniques, such as urine cytology, cystoscopy, and biopsy, detect only a small percentage of low grade NMIBC, due to the difficulty of distinguish low grade cancerous cells from healthy cells [44]. Therefore, new diagnostic strategies for early diagnosis of BC are crucial for proper management, improving the efficacy of treatments and survival rates of patients.

Exosomes are extracellular nanosized vesicles secreted by eukaryotic cells involved in intercellular communication, characterized by lipid bilayers encapsulating DNA, mRNA, microRNA, long non-coding RNA, circular RNA, and proteins. They have a central role in regulating tumor growth, metastasis, and angiogenesis during carcinogenesis, being a potential source of biomarkers. The proteomic analysis of exosomes extracted from cultured bladder cancer cells using sucrose cushion ultracentrifugation was performed by LC-MALDI-TOF/TOF [45]. Hundreds of exosomic proteins were identified and a strong correlation of the observed proteome with carcinoma of bladder and other sites was found using the ExoCarta and Gene Ontology databases. Furthermore, since few clinical specimens were available for the study, only preliminary data relevant to the presence of candidate markers in urinary exosomes from bladder cancer patients were reported. In a study reported by Bryan et al., urine samples collected from 751 BC patients and 127 non-BC subjects were subjected to MALDI proteomic analysis looking for new biomarkers for the pathology and to differentiate between MIBC and NMIBC [46]. Indeed, differences between the two types of BC were observed in proteomic profiles, even if hematuria was significantly more frequent in MIBC cases. Then, the authors eventually observed that, since hematuria is strongly linked to diseases such as BC, the urinary peptidome could be influenced by blood proteins in most patients altering diagnostic data. MALDI-TOF/TOF MS in combination with 2D-DIGE and bioinformatic was also exploited to analyze urine samples collected from bladder cancer patients and healthy subjects trying to improve the early detection rate of bladder cancer and to monitor its evolution [47]. Gc-globulin (GC), a protein involved in the regulation of growth, apoptosis, death, and epidermal growth factor receptor activity, was identified, together with several other proteins all differentially expressed in BC urine samples. The integration of these data with Western blotting, immunohistochemical staining, and enzyme-linked immunosorbent assay (ELISA) quantification of GC (GC-creatinine (Cr) ratio) expression patterns, both in urine and pathological/normal tissues, produced evidence that support the role of GC as a new potential urinary biomarker for the early diagnosis and monitoring of BC. Using a similar approach, the same research group proposed a new urinary biomarker for early detection and monitoring of bladder cancer [48], the protein orosomucoid 1 (ORM1), a transport protein usually involved in immune system modulation during acute-phase reactions, mainly produced by liver, but also by endothelial and some tumor cells. ORM1 was identified in urine among a series of 13 proteins. After verification by Western blotting, ORM1 resulted higher in bladder cancer patients compared to controls and non-malignant cases by ELISA quantification (ORM1-Cr ratio).

In another work, urine samples of pre-operative patients with bladder urothelial neoplasm were pre-treated using two-dimensional gel electrophoresis followed by densitometric analysis. The in-gel tryptic-digested spots were analyzed by MALDI mass spectrometry [49], permitting to identify proteins upregulated (albumin, alpha 1 antitrypsin, apolipoprotein A1, transferrin, transthyretin, haptoglobin and hemoglobin β chain) or downregulated (inter alpha trypsin inhibitor heavy chain) in pathological samples. Moreover, the concentration levels of some of these proteins were associated with the progression of the disease. All the data reported in the work suggest that the analysis of a more significant number of samples could confirm the role of the identified proteins as biomarkers of the disease.

Glycoproteins were the analytical target of a work conducted by Azevedo et al. [50]. They were selectively extracted from urine samples of patients affected by MIBC and low- and high-grade NMIBC using glycan-affinity nanoplatforms, consisting of magnetic nanoprobes coated with three lectins (Concanavalin A, Wheat Germ Agglutinin, and Sambucus nigra). Then, nano-LC MALDI-TOF/TOF analysis and database search allowed the identification of 63 glycoproteins in BC samples only. Furthermore, some glycoproteins were found specifically in low-grade NMIBC and others only in high-grade MIBC, suggesting their potential usefulness for BC early detection and evolution, respectively. It is also worth noting that, for the first time, the presence of CD44, an antigen known to be overexpressed in aggressive BC, was detected in urine samples of high-grade individuals, supporting its potential as a biomarker of BC progression. However, further studies are in progress to identify CD44 isoforms with clinical relevance.

### 2.3. Renal and Lung Cancer

Renal cell carcinoma (RCC) is among the ten most common malignancy for both men and women in Europe [51,52], with predominance in men over women and incidence between 60 and 70 years of age. It is characterized by different histological subtypes, in particular clear cell (ccRCC, 70–80%), papillary (PRCC, 10%), and cromophobe (chRCC, 5%). Smoking, obesity, hypertension, diet, alcohol, and exposure to pollutants are known risk factors for its development, together with hereditary factors. RCC mortality has decreased in recent years due to improved surgical approaches and the development of new drugs. Moreover, various blood and urinary biomarkers for its detection have emerged, even if none of them are able to improve clinical decision-making [53], leaving the search for RCC new diagnostic molecules still an open issue.

With the aim to examine and prioritize peptides whose concentrations could be related to tumor growth measure and clinical data, urine samples of ccRCC patients were pre-fractionated using activated magnetic beads and analyzed by MALDI-TOF [54]. The approach allowed us to obtain the relevant peptide profiles, while further analyses performed by nano-LC–ESI–MS/MS permitted the identification of several peptides. The abundance of some of them turned out to be correlated with the tumor size, stage, and grade. In a work reported by Sandim et al., urine samples from ccRCC patients classified into two distinct grades and healthy donors were analyzed and compared using different strategies to individuate differences in the expressed proteins [55]. At first, protein profiles were evaluated by one dimensional gel electrophoresis coupled to LC-MS/MS. Then, in-solution digestion followed by label-free 1-D LC-MS^E^ and two-dimensional gel electrophoresis coupled to LC-MS/MS and MALDI-TOF/TOF were used for further protein identification. The multiple approach resulted in 354 proteins identified in all samples, allowing us to draw information on proteins differentially expressed in pathological and control cases. A diagnostic strategy using weak cation exchange magnetic bead-based peptide profiling by MALDI-TOF was developed by Dong et al. to detect differential expression of proteins in urine of ccRCC patients and normal controls [56], finding 160 protein peaks that were different between the two sets of samples. In particular, the *m*/*z* ion 2221.71 was found to be significantly different and was proposed as a biomarker for the early detection ccRCC, while the use of genetic algorithms allowed the development of a predictive model of 13 characteristic protein peaks for the diagnosis of the pathology. Magnetic beads separation and MALDI were also successfully used in a further differential proteomic profiling study [57] accomplished on urine samples collected from ccRCC patients, benign kidney diseases patients, and healthy donors, for detecting new biomarkers able to distinguish between benign and malignant masses to be used as an alternative to biopsies.

Lung cancer is the most frequent cancer worldwide, the major cause of death among all types and has a high risk of recurrence [58]. It presents different histological subtypes: non-small cell lung cancer (NSCLC, accounting for about 85% of cases), divided into squamous cell cancer and adenocarcinoma, and small cell lung cancer (SCLC, responsible for the remaining 15%). The 5-year overall survival rate for NSCLC is less than 20% [59] and, as for most tumors, it is deeply influenced by the stage at the time of the diagnosis. Unfortunately, lung cancer usually has a silent onset, most patients ignore the initial symptoms and refuse to undergo expensive and/or invasive conventional diagnostic tests, making the discovery of novel markers for early screening vital.

A significantly different protein expression (22 peptides upregulated and 54 downregulated) between lung adenocarcinoma (LAC) patients and normal controls was found using MALDI-TOF, combined with weak cationic exchange magnetic beads extraction, as an analytical tool for the analysis of the relevant urine samples [60]. Among the 13 most intense detected signals, 12 were identified using nano-LC-MS/MS, while 7 with better diagnostic performances were individuated by receiver operating characteristic analyses. IGKC, AAT, SH3BGRL3, osteopontin, and gelsolin were confirmed overexpressed in LAC by immunohistochemical staining experiments. Indeed, validation using high amounts of samples are needed for the newly identified markers and clinical applications of the procedure are limited, mainly due to high costs and statistical processing requirements. In another work, serum and urine peptides of SCLC patients and healthy individuals were processed using copper ion-chelating nanomagnetic beads and analyzed by MALDI-TOF in combination with ClinProTools, searching for differentially expressed peptides [61]. Some differences in serum and urine peptides between patients with SCLC and healthy individuals were evidenced. Moreover, peptide diagnostic classification models were constructed, showing high sensitivity and specificity.

The protein content of urine samples taken from patients with liver cirrhosis or lung cancer and from healthy donors was investigated using a precipitation procedure followed by SDS electrophoresis and MALDI analysis of the relevant excised and digested bands [62]. Peptide mass fingerprinting identified a 100 kDa protein as the Src substrate cortactin, while various molecular forms of cortactin that differ in the tested groups were identified by Western blot analysis with specific anti-human cortactin antibodies, implying that urine cortactin isoforms could be measured for the diagnosis of acute and systemic diseases.

### 2.4. Other Types of Cancer

Isotope-coded protein labeling and in-solution isoelectric fractionation coupled to LC-MALDI-TOF/TOF were successfully exploited to identify 579 urinary proteins from ovarian cancer patients, searching for potential biomarkers [63]. Some proteins were selected for ESI-Qq-TOF MS analysis to evaluate abundance changes, while the biological relevance of the proteins identified was investigated by Western blot and immunohistochemical analysis by tissue microarray. The approach allowed the identification of both established (e.g., HE4, osteopontin) and novel (e.g., phosphatidylethanolamine binding protein 1, cell-adhesion molecule 1) potential markers for ovarian cancer.

Somatic mosaicism refers to the presence of genetically distinct populations of cells within an individual, a phenomenon that results from the accumulation of somatic mutations occurring during embryonic development and lifetime. Revertant somatic mosaicism is a rare event involving an additional somatic mutation that repairs or compensates a causal mutation. Rather than identifying new biomarkers, Azzollini et al. used MALDI-TOF as a tool to investigate whether revertant somatic mosaicism could be responsible for early onset breast cancer cases among non-carrier relatives of families with BRCA1/2 genes mutations [64], performing a mutation screening on different samples taken from these individuals. Tumor samples (seven cases), blood leukocytes, buccal mucosa and urine samples (two cases), or blood only (seven cases) were analyzed, confirming the absence of the family mutation in all the investigated cases, indicating that a mutation reversion event is unlikely in such patients and that other genetic factors must be involved in these cases.

Zou et al. [65] characterized the glycoconjugate content of exosomes isolated from normal urine samples through multistep differential centrifugation with a view to potential future use of the identified compounds as biomarkers for genitourinary tract diseases. MALDI and LC/MS-MS analyses were independently performed on samples subjected to different pre-treatment approaches and allowed the detection of paucimannosidic, high-mannose, and complex type glycans, the determination of their relative abundances, and the identification of detailed structures. Li et al. combined MALDI-TOF with different sample pre-treatment methods to accomplish a comprehensive screening of phospholipids and lysophospholipids in urine samples, in view of future clinical applications for the detection of various tumors and/or the Alzheimer disease [66], and to perform the proteomic profiling of urine samples collected from gestational trophoblastic neoplasm patients and healthy controls, finding remarkable differences between the groups under investigation [67].

## 3. Saliva Samples Analysis

### 3.1. Oral Cancer

Oral cancer (OC) is the sixth most common cancer with nearly 700,000 new diagnoses reported annually worldwide [68]. Oral squamous cell carcinoma (OSCC), likely formed following premalignant mucosal lesions, is responsible for most of OC cases. OC 5-year survival rate is about 50% and could be improved through early detection, making the development of diagnostic methods alternative to biopsies a key priority.

Higher salivary concentrations of transferrin, also related to tumor size and stage, were found in samples of patients with OSCC compared to controls in a study dedicated to detecting new biomarkers for the disease. [69]. The proteomic profiling was performed by 2-DE followed by MALDI-TOF, while the candidate markers were confirmed by Western blotting, ELISA, and MALDI-TOF/TOF MS. The same research group exploited C8-functionalized magnetic beads to extract peptides from saliva samples of OSCC patients and healthy donors prior to MALDI analysis and ClinProt identification of possible biomarkers [70]. The *m*/*z* ions 2918.57, 5592.64, and 4372.66 were individuated for the discrimination between the two groups of samples. The 24-mer peptide of zinc finger protein 510 (ZNF510) was found only in OSCC samples. These data, integrated with ELISA analysis of saliva and immunohistochemical analysis of tissues, strongly supported the potential of 24-mer zinc finger protein 510 peptide as OSCC salivary biomarker for early detection.

Saliva samples from oral cancer patients and healthy subjects were subjected to electrophoretic separation, in-gel tryptic digestion, and MALDI-TOF-MS/MS analysis to identify suitable tumor marker candidates [71]. A marked proteins overexpression was evidenced in the tumor samples, where annexin 1 and peroxiredoxin 2 were always observed, suggesting their role for the detection of OC, even if a larger group of samples should be processed to fully validate this finding.

Overall, 2-DE and immunoblotting were used on saliva samples of OSCC patients and normal controls by Mu et al. [72]. Some spots were selected for 2-DE image and MALDI analyses followed by database search. The relevant results indicated once again the suitability of differential proteomic approaches for tumor biomarkers discovery.

Since diabetes is a possible risk factor for OSCC development, saliva samples from diabetic and healthy subjects were analyzed using SDS-PAGE and MALDI TOF/TOF mass spectrometry [73]. Annexin A8, Peroxiredoxin-2 levels showed a correlation with the premalignant state type 2 diabetes, implying that their involvement in OSCC development should be considered and confirming the diagnostic value of saliva content. MALDI-TOF/TOF MS was again successfully applied to the analysis of saliva samples to discriminate between different oral diseases, namely oral cancer, oral lichen planus, and chronic periodontitis, searching for specific patterns of proteins mass peaks [74]. FlexAnalysis and ClinProTool were used for quantification. Higher abundances were observed for some *m*/*z* ions in the case of oral cancer (5592.26 and 8301.46) and oral lichen planus (12,964.55 and 13,279.08), while a significant decrease in the *m*/*z* ions 5835.73 and 9801.83 was observed in chronic periodontitis samples. However, the peptide sequences were not identified due to intrinsic instrumental limitations.

The salivary peptidome of T1 stage OSCC patients was compared to that of normal subjects after weak cation exchange magnetic bead extraction followed by MALDI analysis [75], to identify potential tumor markers., Specific *m*/*z* ions were observed in OSCC saliva samples that have higher (1285.6, 1553.5, 1329.9, 1432.2) or lower (1731, 1191.4, 1353.9, 1584.6) relative abundances compared to healthy individuals The *m*/*z* ions 1285.6 and 1432.2 showed the most noticeable differences between the two samples sets and were indicated as putative biomarkers. A study published by Camisasca et al. was devoted to find salivary markers for the detection of oral leukoplakia, a potentially malignant disorder of the oral cavity that could precede carcinoma, using 2-DE after proteins precipitation, in-gel digestion and MALDI-TOF/TOF analysis [76]. Hundreds of proteins were identified in both the sets of samples, while 22 were significantly upregulated in leukoplakia patients, including apolipoprotein A1, alpha amylase, cystatins, keratin 10, and lysozyme precursor. The approach could be useful to test patients at risk of oral cancer for different reasons, such as lifestyle, familiar history, or potential recurrence of a treated oral tumor. Antibody-coupled magnetic beads were also recently reported by Hsiao et al. for the automated immunoenrichment of metalloproteinase-1, a promising salivary biomarker for the diagnosis of OSCC, prior to MALDI analysis [77]. Before enrichment, saliva samples from OSCC patients or normal controls were spotted, dried, and trypsin-digested directly on filter paper. The LOQ was 3.07 ng/mL, and good precision and accuracy were obtained. The approach permitted MMP1 determination in most OSCC samples at concentration levels between 5.95 and 242.52 ng/mL, while it was never detected in the controls. On the contrary, conventional immunoassay was not able to detect the analyte in the considered samples.

### 3.2. Neck Cancer

Head and neck cancers (HNC) are a group of frequent (6% of all) heterogeneous diseases with high incidence of death (1–2% of all) and different self-epidemiology, etiology, and treatment [78]. The most frequent tumor of the group is squamous cell carcinoma (HNSCC, 90% of the cases), whose development is mainly related to tobacco smoke, alcohol, human papillomavirus, and hereditary factors.

Salivary peptide profiles relevant to the analysis of saliva samples from HNC, diabetic, and control subjects were obtained by LC-MALDI-TOF/TOF, with the aim to detect new post-translational modifications on proline-rich proteins [79]. Several glycosylation, phosphorylation, and conversion of Gln to pyro-Glu were individuated, while in HNC samples a prevalence of *N*-acetyl hexosamine modification on basic proline-rich proteins was noted. Another salivary proteomic study was proposed by Jarai et al. for the individuation of new tumor markers for early diagnosis in samples collected from HNSCC patients and normal controls [80]. Protein profiles were obtained by SDS-PAGE followed by MALDI analysis and Mascot database search. Some proteins known to be involved in carcinogenesis were found for the first time, including annexin A1, beta- and gamma-actin, cytokeratin 4 and 13, zinc finger proteins, and P53 pathway proteins. In a lipidomic study reported by Laus et al., saliva samples from HNSCC patients were collected before they underwent a specific organ preservation treatment protocol and analyzed by MALDI-TOF after Bligh–Dyer extraction [81]. Multivariate statistical analyses based on principal component analysis and orthogonal partial least square discriminant analysis was used to find differences between lipid profiles, that allowed us to predict the response to the therapy distinguishing responders from non-responders.

### 3.3. Other Types of Cancer

The connections between the proteins present in saliva samples and the clinical response to a pharmacological treatment were investigated in primary Sjögren’s syndrome and non-Hodgkin’s MALT-type parotid lymphoma patients [82]. Saliva was collected before, during, and 6 months after treatment with cyclophosphamide and rituximab and analyzed by 2-DE and MALDI-TOF/TOF, searching for protein markers whose expression could reflect the clinical status of the patient. Several proteins were identified and exhibited qualitative and quantitative changes, demonstrating the suitability of salivary proteomics in the monitoring of pharmacological treatments outcomes for the pathologies under study. It is likely that, as suggested by the authors, the changes in the salivary glands induced by autoimmune and lymphoproliferative processes might be reflected in saliva proteins. MALDI-TOF/TOF-MS spectra of *N*-glycans were obtained by Qin et al. analyzing saliva samples of different groups of patients [83]. Samples were reduced, alkylated, and trypsin-digested, before incubation with PNGase F and subsequent purification. Among the several *m*/*z* ions attributed to specific *N*-glycans in all samples, 15 were present in all groups, while 3 (2596.925, 2756.962, 2921.031), 2 (1898.676, 1971.692), 5 (1954.677, 2507.914, 2580.930, 2637.952, 3092.120), and 3 (2240.830, 2507.914, 3931.338) were exclusively observed in normal controls, hepatitis B virus-infected patients, cirrhosis patients, and hepatocellular carcinoma patients, respectively. Furthermore, fucosylation and sialylation products seemed to increase and decrease, respectively, in cancer patients. The data obtained from salivary *N*-linked glycome permitted the differentiation of different pathologies and could indicate some possible biomarkers for HCC early diagnosis. Salivary glycans (*N*-and *O*-linked) were also successfully targeted in saliva samples from gastric cancer and atrophic gastritis patients, and healthy individuals [84]. Fucosylated glycoproteins were extracted using Aleuria Aurantia Lectin-magnetic particle conjugates, while *N*- and *O*-linked glycans were enzymatically released and identified by MALDI-TOF/TOF-MS. Several fucosylated *N*-/*O*-linked glycans were found in the analyzed samples, and some of them exhibited increased or decreased expression levels depending on the set of samples, indicating a high potential for diagnostic purposes.

In order to develop a method for the non-invasive early detection of ovarian cancer [85], fluorescence-based 2D-DIGE and MALDI/TOF were used to identify 44 salivary proteins having different expressions, suggesting specific proteins (lipocalin-2, IDO1 and S100A8) as biomarkers for the disease that were also validated by Western blotting and ELISA. Immunohistochemistry was conducted on an independent cohort of ovarian tumor tissues and also demonstrated the overexpression of the putative markers in cancer tissues.

Two recent studies were centered on the search for salivary glycoproteins capable to diagnose breast cancer at early stages, performing MALDI-TOF/TOF analyses of saliva extracts from healthy donors, benign breast cyst patients and breast cancer in stage I/II patients. The results evidenced salivary alterations of galactosylated *N*-/*O*-glycans [86] and *N*-glycans [87], respectively, between the sets of samples analyzed, that could be useful for the identification of new markers for breast cancer. A very recent work [88] reported a MALDI-TOF/TOF method for the detection of *Fusobacterium nucleatum* (*F. nucleatum*) subspecies in saliva samples of patients affected by colorectal cancer and its precursor colorectal adenoma. More than 150 proteins belonging to *F. nucleatum* subspecies were rapidly detected without preculturing, providing precious information to investigate their suspected role in colorectal cancer onset. A new approach for the efficient enrichment of glycopeptides from saliva samples was focused on the development of a stable and reusable covalent organic framework material on which polyethyleneimine, Au nanoparticles, and 4-mercaptophenylboronic acid were successively modified [89]. The use of the new composite material permitted the detection of 56 salivary glycopeptides (31 glycoproteins) by MALDI-TOF and 513 glycopeptides (208 glycoproteins) from the saliva and serum of throat cancer patients by nano-LC-MS/MS.

## 4. Conclusions

Despite the high clinical need, effective tumor biomarkers which could make early diagnosis possible have not yet been established in most cancers, due to the complexity of the task. Biological samples are extremely complex multi-component matrices and potential biomarkers could be present at very low concentrations, especially at the early stage when the diseased tissue is small, hindered by the overwhelming abundance of resident proteins, and rapidly degraded. Additionally, their concentration could vary depending on gender, age, hormonal status, diet, and physical activity of the patients. Even the sample pre-treatment method has a great influence on the observed data and must be selected and optimized for the specific disease under study, meaning that experimental protocols must be standardized. Despite the difficulties of the challenge, the scientific community is continuously making a huge effort in the search for tumor markers. MALDI-TOF mass spectrometry applied to the analysis of non-invasive samples has shown a considerable potential for the search and identification of new biomolecules for the early detection and follow-up of patients of various types of cancer. In the present review of the relevant literature, a significant amount of works, mainly focused on proteomic analysis, with limited lipidomic digressions, have been found. An impressive number of molecules have been identified as potential biomarkers, sometimes proposed as single markers, more often monitoring many potential markers simultaneously, demonstrating how promising the strategy is to reach the goals of precise and early diagnosis of tumor pathologies and easy monitoring of their progression and staging. Moreover, new perspectives could be brought by the progression of molecular subtyping, which could soon lead to a re-elaboration of the data relating to the biomarkers identified over time in light of a new classification system.

## Data Availability

Exclude, no data.
